# Preventive Effects of Avocado/Soybean Unsaponifiables on Complex Regional Pain Syndrome Type I in a Rat Model

**DOI:** 10.3390/biomedicines14020392

**Published:** 2026-02-09

**Authors:** Recep Karasu, Mustafa Dinç, Hünkar Çağdaş Bayrak, Mehmet Emre Topçu

**Affiliations:** 1 Department of Orthopedics and Traumatology, Bursa City Hospital, 16250 Bursa, Turkey; recepkarasu@hotmail.com (R.K.); drindianster@gmail.com (M.D.); 2 Department of Orthopedics and Traumatology, Bursa Yüksek İhtisas Research Hospital, University of Health Sciences, 16310 Bursa, Turkey; 3 Department of Pathology, Faculty of Veterinary Medicine, Uludag University, 16059 Bursa, Turkey; mehmetemretopcu@uludag.edu.tr

**Keywords:** Complex Regional Pain Syndrome Type I (CRPS-I), avocado/soybean unsaponifiables (ASU), fracture-immobilization model, neuroinflammation, oxidative stress

## Abstract

**Background and Object:** Complex Regional Pain Syndrome Type I (CRPS-I) is a debilitating condition often triggered by trauma, with early pathophysiology driven by neuroinflammation and oxidative stress. Avocado/soybean unsaponifiables (ASU) possess potent anti-inflammatory and antioxidant properties but have never been tested for CRPS-I prevention. This study investigated the preventive effects of early systemic administration of ASU on the development of CRPS-I-like features in a validated rat model of tibial fracture and cast immobilization. **Methods:** Twenty adult male Wistar rats were randomized into two groups (*n* = 10/group): a CRPS-I (Vehicle) group receiving daily intraperitoneal saline, and a CRPS-I+ASU group receiving daily ASU (300 mg/kg/day). The model was induced via a right tibial fracture followed by 28 days of cast immobilization. Treatment began immediately post-fracture. Behavioral outcomes (mechanical allodynia via von Frey, paw edema, temperature asymmetry) were assessed pre-fracture and on day 29. Subsequently, levels of pro-inflammatory cytokines (IL-1β, IL-6, TNF-α) and oxidative stress markers (TAS, TOS, OSI) were measured in the ipsilateral hind paw tissue. **Results:** ASU treatment significantly attenuated the development of CRPS-I-like manifestations. Compared to the vehicle group, the ASU group exhibited a markedly lower median percentage decrease in mechanical withdrawal threshold (30.20% [22.56–37.01] vs. 51.45% [47.84–61.11], *p* = 0.001), reduced temperature asymmetry (0.75 °C [0.55–1.00] vs. 1.95 °C [1.80–2.33], *p* < 0.001), and less paw edema (8.35% [7.06–11.29] vs. 14.75% [12.66–19.20], *p* = 0.004). Biochemically, ASU treatment significantly suppressed tissue levels of IL-1β, IL-6, and TNF-α (all *p* < 0.001), enhanced total antioxidant status (TAS), and reduced total oxidant status (TOS) and the oxidative stress index (OSI) (all *p* < 0.001). **Conclusions:** Early systemic administration of ASU significantly prevents the development of nociceptive, vascular, inflammatory, and oxidative disturbances in a rat model of CRPS-I. These findings highlight ASU’s multimodal protective effects at the tissue level and position it as a promising candidate for early preventive intervention in post-traumatic CRPS-I.

## 1. Introduction

Complex regional pain syndrome type I (CRPS-I) is a debilitating pain condition that typically develops after minor trauma, fractures, or immobilization, and is characterized by disproportionate pain, sensory disturbances, vasomotor changes, edema, and trophic alterations [1]. Although its clinical burden is significant, the underlying pathophysiology remains incompletely understood, and effective early-phase preventive therapies are still lacking [2].

Accumulating evidence indicates that the early phase of CRPS-I is driven by a convergence of neurogenic inflammation, peripheral and central sensitization, microvascular dysfunction, and oxidative–nitrosative stress [3,4]. Elevated levels of pro-inflammatory cytokines—particularly IL-1β, IL-6, and TNF-α—along with chemokines such as CCL2 and CXCL1, contribute to neuro–immune crosstalk, leukocyte recruitment, and glial activation, thereby facilitating nociceptor sensitization and sustained inflammatory amplification [5,6,7,8,9,10,11]. These inflammatory processes are further regulated by upstream signaling pathways, including aberrant activation of Wnt signaling, which has been shown to enhance neuroinflammation and pain sensitization in chronic pain states [12]. Concurrently, increased production of reactive oxygen species (ROS) and impaired antioxidant capacity further promote nociceptor hyperexcitability, endothelial dysfunction, and persistent pain behavior [5,6,7,8]. These interconnected molecular pathways underscore the importance of early modulation to prevent progression to chronic CRPS-I.

Given the central role of oxidative stress and inflammation in CRPS-I pathophysiology, several antioxidant and anti-inflammatory agents have been investigated for their preventive potential. For example, vitamin C has been shown to reduce mechanical allodynia, edema, and oxidative damage in animal models and has demonstrated prophylactic benefits in clinical fracture settings [13,14,15]. Similarly, α-lipoic acid and N-acetylcysteine have been shown to attenuate hyperalgesia, suppress pro-inflammatory cytokine production and neuroinflammation, and reduce oxidative stress in experimental models relevant to CRPS-I, including ischemia–reperfusion and fracture-immobilization paradigms [16,17,18]. Collectively, these findings emphasize that early targeting of oxidative and inflammatory pathways may be an effective strategy for reducing CRPS-I severity after trauma.

To experimentally evaluate this strategy of early pathway modulation, well-established rodent models of CRPS-I, such as tibial fracture followed by cast immobilization, are invaluable. These models successfully reproduce hallmark clinical features including mechanical allodynia, edema, temperature asymmetry, neurogenic inflammation, and early oxidative stress responses [19,20,21,22,23]. This robust and widely validated paradigm allows for the controlled investigation of CRPS-I pathophysiology and facilitates the preclinical evaluation of potential preventive interventions.

Among the candidate agents targeting these pathways, avocado/soybean unsaponifiables (ASU)—a natural compound composed of one-third avocado oil and two-thirds soybean oil—represent a promising yet unexplored option. ASU is known for its anti-inflammatory, antioxidant, and chondroprotective effects [24,25]. Experimental and clinical studies, particularly in osteoarthritis, have consistently shown that ASU significantly reduces the production of key pro-inflammatory cytokines—including IL-1β, IL-6, and TNF-α—and decreases oxidative stress markers, supporting its role as a biological modulator of inflammatory pathways [25,26]. These mechanistic effects directly overlap with the early pathophysiological processes observed in CRPS-I, which are characterized by pronounced cytokine activation and oxidative–nitrosative stress. However, despite this strong pharmacological rationale, the potential preventive impact of ASU on trauma-induced inflammation and oxidative stress during the early post-injury phase of CRPS-I has not yet been investigated.

Based on this mechanistic overlap, we hypothesized that systemic ASU administration initiated immediately after fracture, prior to the establishment of chronic CRPS-I would attenuate nociceptive hypersensitivity, inflammatory cytokine release, and oxidative stress, thereby reducing progression toward CRPS-I in a relevant animal model. To test this hypothesis, we employed a validated rat model involving tibial fracture followed by cast immobilization and compared untreated controls with ASU-treated animals. Behavioral, inflammatory, and oxidative stress markers were assessed to determine the preventive efficacy of ASU. To our knowledge, this study is the first to evaluate the preventive effects of ASU on CRPS-I development.

## 2. Materials and Methods

### 2.1. Study Design and Ethical Approval

This controlled laboratory experiment was designed based on established CRPS-I rodent research standards, which emphasize fracture-induced neuroinflammation and immobilization-driven sensitization as key model components [19,27]. The study protocol received full approval from the Uludağ University Animal Experiments Local Ethics Committee (HADYEK) (Approval No: 2025-09/03; Date: 1 July 2025). All procedures were conducted in strict accordance with the National Institutes of Health Guide for the Care and Use of Laboratory Animals [28] and reported following the ARRIVE 2.0 guidelines [29]. The ethical approval process ensured adherence to the 3Rs principle (Replacement, Reduction, Refinement) [30], justified the sample size based on a priori power analysis [31] and mandated predefined humane endpoints [32].

### 2.2. Animals and Housing Conditions

Twenty adult male Wistar albino rats (10–12 weeks old; initial weight 300–350 g) were used in this study. The use of male rats was to avoid the confounding hormonal variability associated with the estrous cycle in females, a standard approach in initial mechanistic studies of this model [33,34]. Animals were sourced from the Uludağ University Experimental Animal Research and Breeding Center, a specific pathogen-free (SPF) facility. Rats were pair-housed in standard polycarbonate cages with corncob bedding under controlled environmental conditions: temperature 22 ± 2 °C, humidity 55 ± 5%, and a 12 h light/dark cycle (lights on at 07:00) [28,35]. Standard pelleted chow and water were available ad libitum throughout the experiment. All animals were acclimatized to the housing facility for one week prior to any procedures [35].

### 2.3. Randomization, Blinding, and Experimental Groups

Following acclimatization, rats were randomly assigned to one of two experimental groups (*n* = 10 per group) using a computer-generated block randomization sequence (GraphPad QuickCalcs) to minimize selection bias [31,36]. The group allocation was concealed from the researcher performing the surgeries and all subsequent behavioral and biochemical analyses (double-blind design) to mitigate assessment bias [37]. Daily injections were administered by a separate researcher who was not involved in any outcome assessments. The experimental groups were:

- CRPS-I (Vehicle) Group: Underwent tibial fracture + cast immobilization and received daily intraperitoneal (i.p.) injections of sterile 0.9% saline (vehicle).
- CRPS-I + ASU Group: Underwent tibial fracture + cast immobilization and received daily i.p. injections of ASU (300 mg/kg/day) suspended in sterile 0.9% saline.

### 2.4. Tibial Fracture and Cast Immobilization Procedure

The CRPS-I model was induced using the well-characterized tibial fracture with cast immobilization method [19,27]. Briefly, general anesthesia was induced using 5% sevoflurane in an induction chamber and maintained with 2–3% sevoflurane delivered via a nose cone during the operation, a standard inhalational protocol providing rapid induction and stable surgical plane anesthesia in rodents [32,38]. The depth of anesthesia was confirmed by the absence of pedal and corneal reflexes. Under aseptic conditions, a closed mid-diaphyseal fracture of the right tibia was produced manually using a three-point bending technique, ensuring a simple fracture without comminution or skin penetration, as described in the original model protocol [27].

Immediately after fracture, the affected hindlimb was wrapped in a soft cotton bandage, and a lightweight fiberglass cast (Scotchcast™, 3M, Alexandria, VA, USA) was applied from the metatarsophalangeal joints to a point just proximal to the knee. The ankle was maintained in a plantar-flexed position (approximately 90°), and the toes were left exposed to monitor circulation and prevent ischemia—a critical step to avoid secondary neurovascular compromise [39]. The contralateral limb remained free.

Postoperative analgesia was initiated immediately upon recovery from anesthesia via subcutaneous buprenorphine (0.05 mg/kg) administered every 8–12 h for the first 48 h, in accordance with established recommendations for multimodal pain management in rodent surgery [32,40]. Animals were monitored at least twice daily for the first 72 h, and then daily thereafter, for signs of distress, weight loss (>20%), autotomy, or cast complications, following established welfare assessment protocols. The cast remained in place for 28 days, a duration standardized to produce persistent CRPS-I-like behavioral and inflammatory changes [19,27,39].

### 2.5. Drug Preparation and Administration

Avocado/soybean unsaponifiables (ASU) was obtained as a standardized pharmaceutical-grade formulation (Piascledine^®^ 300, Laboratoires Expanscience, Paris, France). For daily intraperitoneal administration, the ASU powder was freshly suspended in sterile 0.9% saline to achieve a concentration of 60 mg/mL. The suspension was homogenized by vortexing for 1 min followed by sonication for 30 s. Rats in the treatment group received ASU at a dose of 300 mg/kg/day (injection volume: 5 mL/kg), while the vehicle control group received an equivalent volume of saline alone. The first injection was administered immediately after fracture and cast application (postoperative day 1), and treatment continued once daily for the entire 28-day immobilization period. This administration schedule was specifically designed to evaluate the preventive effects of ASU during the early post-fracture phase, rather than its therapeutic efficacy in established CRPS-I.

The selected dose of 300 mg/kg/day was based on previous preclinical studies demonstrating significant anti-inflammatory and antioxidant efficacy at this dosage [26,41,42,43]. Administration was initiated on postoperative day 1 to target the early peak of pro-inflammatory cytokines and oxidative stress that are critical for the establishment of chronic CRPS-I pathology, as established in the foundational literature of this fracture model [19,22,27,39,44,45].

Injections were repeated once daily at the same time each morning for the entire 28-day immobilization period. The intraperitoneal route was chosen for reliable and consistent systemic delivery, ensuring high bioavailability to modulate both peripheral and central inflammatory pathways. This route is a standard and well-validated method for administering lipid-soluble compounds and anti-inflammatory agents in rodent pain research [46,47,48,49,50,51]. The injection volume was standardized at 5 mL/kg, which is within the recommended safe volume for intraperitoneal administration in rats [52,53].

The vehicle control group received an equivalent volume of sterile 0.9% saline alone, administered on the same schedule.

### 2.6. Behavioral Assessments

Behavioral tests were conducted by an experimenter blinded to the group allocation. All animals were habituated to the testing apparatus for 20 min on two consecutive days prior to baseline measurements. Assessments were performed at baseline (pre-fracture) and 24 h after cast removal (Day 29).

### 2.7. Mechanical Allodynia (Von Frey Test)

Mechanical withdrawal thresholds of the hind paws were assessed using the up-down method with a calibrated series of von Frey filaments (0.02, 0.04, 0.07, 0.16, 0.4, 0.6, 1.0, 1.4, 2.0 g; Stoelting Co., Wood Dale, IL, USA) [54,55]. Rats were placed in individual clear acrylic chambers on an elevated wire mesh grid and acclimated for 15–20 min before testing. Filaments were applied perpendicularly to the plantar surface of the ipsilateral (fractured) hind paw until bending and held for 3–4 s. A positive response was noted as a sharp paw withdrawal, shaking, or licking. The 50% mechanical paw withdrawal threshold (PWT) was calculated using the Dixon formula. To account for baseline variability between animals, the results were expressed as the percentage decrease from the pre-fracture baseline, calculated as follows:

% Decrease = [(Baseline PWT − Post-immobilization PWT)/Baseline PWT] × 100.

### 2.8. Paw Edema Measurement

Hind paw edema was quantified as the difference in dorsal-plantar thickness between the fractured (ipsilateral) and non-fractured (contralateral) limbs, a standard method for assessing inflammation in rodent fracture models [19,27]. Measurements were taken using a digital caliper (Mitutoyo, Kawasaki, Japan, precision 0.01 mm) at the level of the metatarsals. The percentage increase in paw thickness was calculated as: [(Ipsilateral thickness − Contralateral thickness)/Contralateral thickness] × 100.

### 2.9. Hind Paw Temperature Measurement

The skin temperature of the plantar surface of both hind paws was measured using a non-contact infrared thermometer (FLIR ONE Pro, FLIR Systems, Wilsonville, OR, USA; accuracy ±0.1 °C). To ensure measurement consistency, the room temperature was stabilized at 23 ± 1 °C, and rats were acclimated in the testing room for 30 min prior to measurement. During measurement, rats were gently restrained, and the thermometer was held perpendicular to the plantar surface at a standardized distance of approximately 2–3 cm. Three consecutive readings were taken for each paw at 1 min intervals, and the mean value was recorded. Temperature asymmetry, indicative of vasomotor dysfunction, was calculated as the difference (°C) between the ipsilateral (fractured) and contralateral (non-fractured) paws (ΔT = T_ipsilateral − T_contralateral).

### 2.10. Sample Collection and Preparation

On Day 29, after the final behavioral tests, rats were deeply anesthetized. Following confirmation of surgical plane anesthesia, a standardized full-thickness skin and subcutaneous tissue sample (approximately 1 cm^2^) was aseptically harvested from the plantar surface of the ipsilateral (fractured) hind paw. Animals were subsequently euthanized by exsanguination while under deep anesthesia. Tissue samples were immediately snap-frozen in liquid nitrogen and stored at −80 °C until biochemical analysis.

For tissue homogenization, frozen samples were weighed and homogenized on ice in 10 volumes (*w*/*v*) of cold radioimmunoprecipitation assay (RIPA) buffer supplemented with 1% (*v*/*v*) protease and phosphatase inhibitor cocktail (Thermo Fisher Scientific, Waltham, MA, USA) using a mechanical homogenizer. The homogenates were centrifuged at 12,000× *g* for 20 min at 4 °C. The supernatant was carefully collected, and the total protein concentration was determined using a bicinchoninic acid (BCA) assay kit (Pierce™, Thermo Fisher Scientific, USA) according to the manufacturer’s instructions, with bovine serum albumin (BSA) as the standard [56]. All tissue lysate results were normalized to the total protein content and expressed as picograms (pg) or units (U) per milligram (mg) of protein.

### 2.11. Biochemical Analyses

#### 2.11.1. Inflammatory Cytokine Measurement

The concentrations of interleukin-1β (IL-1β), interleukin-6 (IL-6), and tumor necrosis factor-alpha (TNF-α) in tissue homogenates were quantified using commercially available, rat-specific enzyme-linked immunosorbent assay (ELISA) kits (Elabscience^®^, Wuhan, China; Cat# E-EL-R0012 for IL-1β, E-EL-R0015 for IL-6, and E-EL-R2856 for TNF-α). All assays were performed strictly in accordance with the manufacturer’s instructions. Briefly, standards and samples were added to the pre-coated wells, followed by the respective detection antibodies and enzyme conjugate. After incubation and washing steps, the substrate solution was added. The reaction was stopped, and the absorbance was immediately measured at 450 nm with a reference wavelength of 570 nm using a microplate reader (BioTek Epoch 2, Agilent, Santa Clara, CA, USA). The intra- and inter-assay coefficients of variation were below 10%

#### 2.11.2. Oxidative Stress Markers Measurement

Oxidative stress in tissue homogenates was evaluated by measuring the total antioxidant status (TAS) and total oxidant status (TOS) using fully automated, colorimetric commercial kits (Rel Assay Diagnostics, Gaziantep, Turkey). These assays are based on the methods originally described by Erel [57,58]. The assays were performed on tissue homogenates according to the manufacturer’s instructions for liquid samples. The obtained values (in mmol Trolox equivalent/L for TAS and μmol H_2_O_2_ equivalent/L for TOS) were then normalized to the total protein concentration of each homogenate, as determined by the BCA assay. The oxidative stress index (OSI), a composite marker reflecting the overall redox balance, was calculated using the following formula: OSI (arbitrary unit) = $\left(TOS,\mu molH_{2}O_{2}Equiv./mgprotein)/(TAS,mmolTroloxEquiv./mgprotein\right)$ × 100.

### 2.12. Statistical Analysis

All statistical analyses were performed using IBM SPSS Statistics version 27.0 (IBM Corp., Armonk, NY, USA). Sample size determination followed the research equation method, which is widely recommended for controlled animal experiments where prior variance estimates are limited and ethical considerations require minimizing animal use. Accordingly, equal group sizes were adopted to ensure sufficient sensitivity for detecting biologically relevant differences while maintaining experimental balance.

Data distribution was evaluated using the Shapiro–Wilk test and visual inspection of Q–Q plots. As most variables did not meet normality assumptions, non-parametric statistical methods were applied. Continuous variables are presented as median (25th–75th percentile) and mean ± standard deviation (SD) to facilitate both robust inference and comparability with prior experimental pain and neuroinflammation studies.

Between-group comparisons were performed using the Mann–Whitney U test, which is appropriate for small-sample animal studies with skewed distributions. For outcomes demonstrating statistically significant differences, the magnitude of group effects was further quantified using the non-parametric effect size (r), calculated as *r = Z/√N*, where *Z* represents the standardized test statistic and *N* the total sample size. Post hoc evaluation revealed that effect sizes for significant outcomes were consistently large, supporting the biological relevance of the observed differences.

All statistical tests were two-tailed, and a *p*-value < 0.05 was considered statistically significant. No data points were excluded, and no imputation procedures were applied. All analyses were conducted blinded to group allocation to minimize observer bias.

## 3. Results

### 3.1. General Observations

All twenty animals (*n* = 10 per group) completed the full 4-week immobilization protocol without postoperative complications, cast-related ischemia, autotomy, or protocol deviations. No mortality occurred. Body weight and general health status were comparable between groups throughout the study period.

### 3.2. Behavioral and Physiological Outcomes

#### 3.2.1. Mechanical Allodynia Results (Von Frey Test)

Following 28 days of cast immobilization, the mechanical withdrawal threshold of the ipsilateral hind paw decreased in both groups. The median percentage decrease from baseline was 51.45% [47.84–61.11] in the vehicle-treated control group, compared to 30.20% [22.56–37.01] in the ASU-treated group (*p* = 0.001, r = 0.78) (Table 1).

#### 3.2.2. Hind Paw Temperature Difference

The temperature asymmetry between the fractured (ipsilateral) and contralateral hind paws was significantly greater in the control group (1.95 [1.80–2.33] °C) than in the ASU-treated group (0.75 [0.55–1.00] °C) (*p* < 0.001, r = 0.84) (Table 1).

#### 3.2.3. Paw Edema (%)

**Figure 1:**
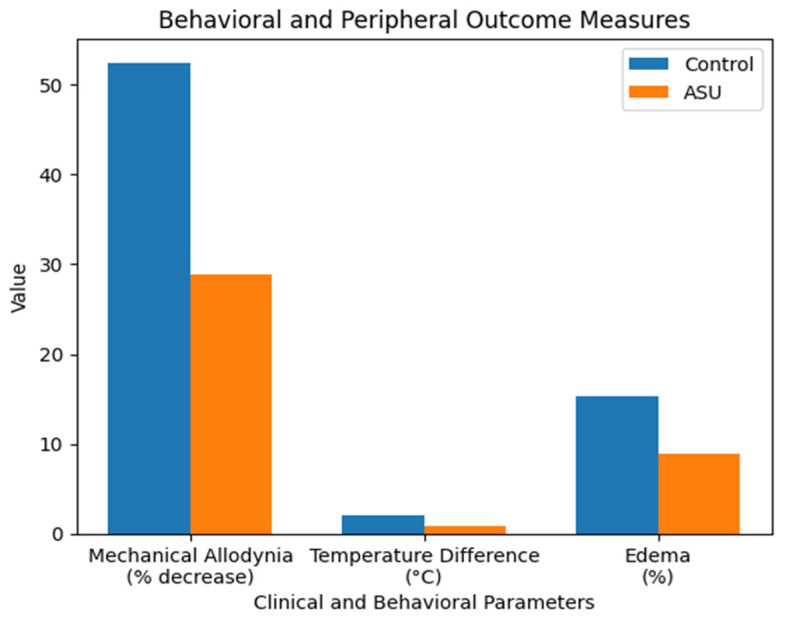
Effects of ASU on behavioral and peripheral outcome measures. Bar graphs illustrating mechanical allodynia (% decrease, Von Frey test), temperature difference (°C), and edema (%) in Control and ASU-treated groups. Data are presented as mean values. ASU treatment significantly improved all parameters compared with controls.

The percentage increase in paw thickness (edema) was greater in the control group (14.75 [12.66–19.20] %) than in the ASU-treated group (8.35 [7.06–11.29] %) (*p* = 0.004, r = 0.65) (Table 1) (Figure 1).

### 3.3. Inflammatory Cytokine Levels in Paw Tissue

**Figure 2:**
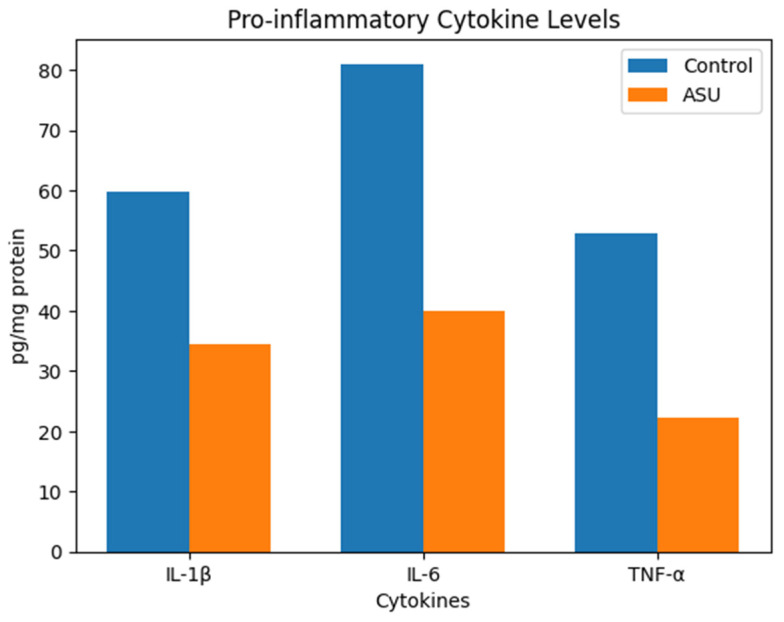
Effects of ASU on pro-inflammatory cytokine levels in ipsilateral Hind Paw Tissue Homogenates. Bar graphs showing tissue levels of IL-1β, IL-6, and TNF-α (pg/mg protein) in Control and ASU-treated groups. Data are presented as mean values. All cytokines were significantly reduced in the ASU group compared with controls (*p* < 0.001).

The concentrations of pro-inflammatory cytokines in ipsilateral hind paw tissue homogenates were quantified. IL-1β levels were 34.90 [31.13–37.09] pg/mg protein in the ASU group compared to 59.84 [52.32–65.20] pg/mg protein in the control group (*p* < 0.001, r = 0.84). IL-6 levels were 40.25 [36.04–44.15] pg/mg protein in the ASU group versus 82.39 [74.29–91.51] pg/mg protein in controls (*p* < 0.001, r = 0.84). TNF-α levels were 22.12 [19.82–24.35] pg/mg protein in the ASU group versus 52.02 [48.84–57.85] pg/mg protein in controls (*p* < 0.001, r = 0.84) (Table 2) (Figure 2).

### 3.4. Oxidative Stress Parameters in Paw Tissue

**Figure 3:**
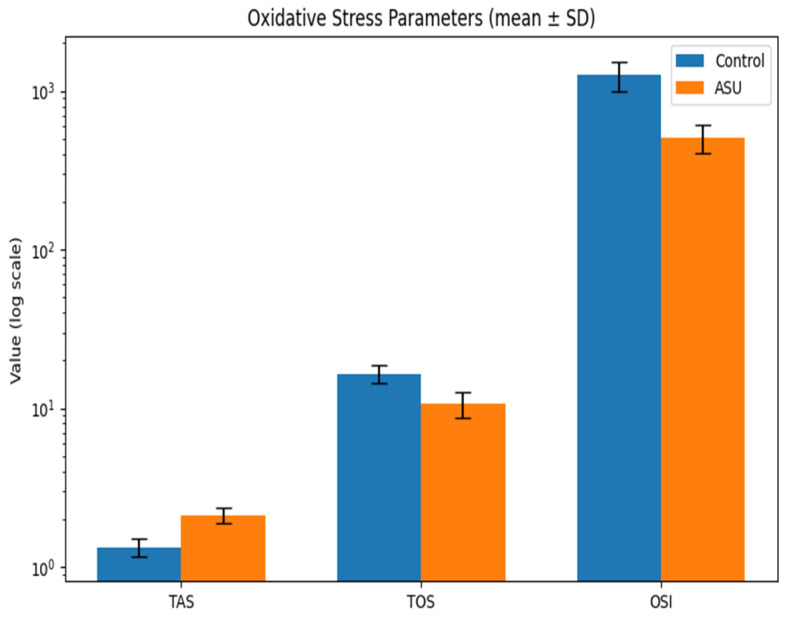
Effects of ASU on oxidative stress parameters in Ipsilateral Hind Paw Tissue Homogenates. Bar graph showing tissue-level total antioxidant status (TAS, mmol Trolox equivalents per mg protein), total oxidant status (TOS, µmol H_2_O_2_ equivalents per mg protein), and oxidative stress index (OSI, arbitrary units) in Control (blue) and ASU-treated (orange) groups. Data are presented as mean ± standard deviation and plotted on a logarithmic scale to allow visualization of parameters with different magnitudes. ASU treatment significantly increased TAS while significantly reducing TOS and OSI compared with controls (all *p* < 0.001).

Total antioxidant status (TAS) in paw tissue homogenates was higher in the ASU group (2.06 [1.92–2.29] mmol Trolox equation/mg protein) than in the control group (1.36 [1.16–1.40] mmol Trolox equation/mg protein) (*p* < 0.001, r = 0.84). Total oxidant status (TOS) was lower in the ASU group (11.20 [8.46–12.03] µmol H_2_O_2_ equation/mg protein) compared to controls (17.32 [14.15–17.77] µmol H_2_O_2_ equation/mg protein) (*p* < 0.001, r = 0.80). The oxidative stress index (OSI), calculated as (TOS/TAS) × 100, was significantly lower in ASU-treated animals (488.49 [425.91–605.11] AU) than in controls (1272.18 [973.90–1531.90] AU) (*p* < 0.001, r = 0.84) (Table 3) (Figure 3).

## 4. Discussion

The present study demonstrates for the first time that early systemic administration of avocado/soybean unsaponifiables (ASU) substantially attenuates the development of CRPS-I-like features in a validated tibial fracture–cast immobilization model [19,27,44]. By concurrently improving behavioral hypersensitivity, vascular alterations, and biochemical indices of inflammation and oxidative stress, ASU appears to act on multiple interrelated biological pathways that determine early CRPS-I progression [3,4,8]. These multidimensional effects position ASU as a promising preventive strategy targeting the acute phase following limb trauma and immobilization. Notably, the observed effects were associated with large effect sizes (r > 0.65), suggesting a robust biological impact beyond mere statistical significance.

Interpreting the behavioral findings in the context of CRPS-I pathophysiology provides important mechanistic insight. Mechanical allodynia in CRPS-I arises from intensified nociceptor responsiveness and enhanced spinal neuroimmune signaling [3,5,19,22,39]. Consistent with prior fracture–immobilization studies, in our study vehicle-treated animals displayed pronounced reductions in withdrawal thresholds indicative of sensitization [19,21,27,59]. In contrast, ASU-treated rats exhibited significantly milder hypersensitivity, suggesting that ASU interrupts the early inflammatory and oxidative milieu that otherwise promotes nociceptor hyperexcitability. The concurrent reduction in paw edema and temperature asymmetry further supports ASU’s ability to stabilize neurovascular and inflammatory responses—phenomena closely linked to early CRPS-I features such as vasomotor imbalance and neurogenic inflammation [2,4,19,23,60].

This behavioral improvement is strongly supported by the biochemical profile. ASU produced a marked suppression of IL-1β, IL-6, and TNF-α, cytokines that orchestrate vascular reactivity, peripheral sensitization, and glial activation in CRPS-I [5,6,39]. These findings align with established reports demonstrating that ASU markedly reduces pro-inflammatory cytokine production in chondrocytes, synoviocytes, and multiple inflammatory models [25,26,60,61]. More importantly, extending these anti-inflammatory properties to a trauma-induced neuroinflammatory condition underscores ASU’s broader mechanistic relevance beyond joint disease. Parallel to its cytokine-modulating effects, ASU substantially improved tissue level oxidative balance by elevating TAS and reducing TOS and OSI, consistent with previous studies documenting its antioxidant properties [24,62]. Because oxidative stress amplifies cytokine signaling, promotes endothelial dysfunction, and sensitizes nociceptors in CRPS-I [3,4,8], restoring redox homeostasis likely represents a central mechanism through which ASU confers its protective effects. The coordinated reduction in cytokines and oxidative stress may disrupt a vicious cycle where each potentiates the other, ultimately preventing the establishment of a chronic pain state characterized by peripheral and central sensitization.

Comparison with previously investigated CRPS-I interventions contextualizes the mechanistic profile of ASU. Vitamin C remains the most clinically established prophylactic agent, acting primarily through its antioxidant capacity to mitigate oxidative stress and endothelial dysfunction, with some studies also reporting modest anti-inflammatory effects; its ability to reduce CRPS-I incidence after distal radius fractures is well-supported [13,14,15]. N-acetylcysteine (NAC) has demonstrated reductions in both oxidative stress markers and pro-inflammatory cytokines (IL-1β, IL-6) in recent fracture–immobilization models, accompanied by improvements in nociceptive thresholds. However, the magnitude and consistency of its behavioral effects across earlier clinical and preclinical studies have been variable [17,18]. α-Lipoic acid exerts strong antioxidant and mitochondrial-protective actions, and emerging evidence suggests that it can also attenuate neuroinflammation and reduce selected cytokines (e.g., TNF-α, IL-1β) in neuropathic pain models, including CRPS-I [16]. Targeted anti-inflammatory therapies—such as corticosteroids or IL-1 receptor antagonists—effectively downregulate cytokine-driven pathways but generally do not address the concurrent oxidative imbalance characteristic of early CRPS-I, which may limit their preventive utility when used in isolation. Within this preventive context, the present study positions ASU as another multi-target agent relevant to early CRPS-I pathophysiology. ASU elicited a robust and simultaneous suppression of key pro-inflammatory cytokines (IL-1β, IL-6, TNF-α) together with shift toward a more favorable tissue level redox balance, yielding consistent preventive effects across behavioral, vascular, and biochemical domains. This broad yet physiologically coherent effect profile aligns with the interconnected inflammatory and oxidative mechanisms that define early CRPS-I [3,4,8]. Although ASU is not “unique” in its dual targeting, the magnitude, consistency, and cross-domain relevance of its effects in this model underscore its distinct promise as a preventive candidate warranting further translational evaluation.

While these findings provide strong evidence supporting ASU’s preventive efficacy, several limitations merit discussion. First, the lack of a sham-operated control group (undergoing anesthesia and surgical exposure without fracture and immobilization) limits our ability to conclude whether ASU specifically reverses pathology or also modulates baseline physiology. Without a non-injured baseline reference, we cannot definitively state that the observed reductions in pro-inflammatory cytokines and oxidative stress markers represent a “normalization” or “restoration” to physiological levels, rather than a significant attenuation of the injury-induced elevations. The inclusion of such a group in future studies is crucial to precisely define ASU’s preventive effect. Second, it should be emphasized that the present study was not designed to delineate specific intracellular signaling pathways. While the observed reductions in pro-inflammatory cytokines and oxidative stress markers are consistent with known actions of ASU, key molecular mediators implicated in CRPS-I pathophysiology—such as NF-κB, Nrf2, MAPK signaling cascades, COX-2, or iNOS—were not directly assessed [3,4,8]. Therefore, the current findings should be interpreted as a functional validation of ASU’s biological effects in a CRPS-I–relevant model rather than a definitive mechanistic elucidation of its molecular targets. Further pathway-focused studies will be required to clarify whether ASU primarily modulates inflammatory initiation, amplification, or downstream effector processes. Third, the use of only male animals limits extrapolation, given sex-related differences in immune and nociceptive responses. Future studies should include both sexes to evaluate the generalizability of these promising findings. Fourth, our biochemical analysis was confined to peripheral paw tissue. Examinations of dorsal root ganglia or spinal cord markers would be necessary to clarify the central contributions to ASU’s mechanism of action in future studies. Fifth, only a single ASU dose was evaluated, and pharmacokinetic data in the context of acute trauma are lacking. Furthermore, the study design focused on a single post-immobilization time point (Day 29). The lack of intermediate time points during the 4-week immobilization period prevents analysis of the temporal dynamics of ASU’s effects (e.g., whether it halts or merely delays progression), and the absence of post-cast-removal follow-up assessments limits conclusions about the durability of its benefits and whether ASU truly prevents disease progression versus transiently attenuates severity at this specific stage. Another limitation, the study did not include histopathological evaluation of the affected paw tissue. While our biochemical data strongly indicate reduced inflammation and oxidative stress, direct visual evidence from histology (e.g., assessment of inflammatory cell infiltration, dermal thickness, nerve fiber morphology, or vascular changes) is lacking. Incorporating such analyses in future studies would provide crucial structural correlates to the molecular and behavioral findings. Finally, although the fracture–immobilization model is highly translational for post-traumatic CRPS-I, CRPS arising from alternative mechanisms (e.g., ischemia–reperfusion injury) may differ in their responsiveness to ASU.

Despite these limitations, the clinical implications of these findings are compelling. Because early cytokine activation and oxidative–nitrosative stress strongly predict CRPS-I development, interventions that modulate these processes immediately after injury may meaningfully reduce chronic progression. Importantly, these implications relate to early post-injury intervention rather than treatment of established CRPS-I. ASU’s well-established safety profile, widespread clinical use, and oral availability provide a practical foundation for translational exploration in acute fracture care. The dual modulation of inflammatory and oxidative pathways suggests that ASU may influence not only symptom severity but also the biological trajectory leading to chronic CRPS-I.

Future research should aim to refine and extend these findings. Dose–response and timing studies are needed to identify optimal administration strategies. Mechanistic investigations incorporating spinal and supraspinal markers would clarify central contributions to ASU’s effects. Longitudinal studies evaluating post-immobilization recovery will determine whether ASU prevents chronic CRPS-like outcomes. Finally, translational studies in fracture patients at elevated risk for CRPS-I could establish feasibility and guide the design of future randomized clinical trials. Given the relatively low incidence of CRPS-I, such translational efforts will also be essential to define which patient subgroups may derive the greatest benefit from preventive interventions such as ASU.

## 5. Conclusions

In conclusion, this study demonstrates that early systemic ASU administration significantly attenuates pain behavior, vascular alterations, and key inflammatory and oxidative markers in a validated model of CRPS-I. The consistent, large-magnitude effects across behavioral, vascular, and molecular domains underscore ASU’s potential as a multifaceted preventive agent. These results highlight ASU as a biologically rational, mechanistically comprehensive, and clinically accessible candidate for early CRPS-I prevention, rather than for treatment of established disease and provide a strong foundation for future preclinical and clinical investigations.

## Figures and Tables

**Table 1:** Behavioral and Physiological Parameters Measured 24 Hours After Cast Removal Following 4 Weeks of Immobilization.

Parameter	Control (Median [IQR]; Mean ± SD)	ASU (Median [IQR]; Mean ± SD)	*p*-Value	Effect Size (r)
Von Frey (% decrease)	51.45 [47.84–61.11]; 52.53 ± 9.59	30.20 [22.56–37.01]; 2 8.94 ± 10.50	0.001	0.78
Temperature difference (°C)	1.95 [1.80–2.33]; 2.06 ± 0.37	0.75 [0.55–1.00]; 0.76 ± 0.32	<0.001	0.84
Edema (%)	14.75 [12.66–19.20]; 15.23 ± 4.79	8.35 [7.06–11.29]; 8.92 ± 2.50	0.004	0.65

Data are presented as median [interquartile range, IQR] and mean ± standard deviation (SD). Group comparisons were performed using the Mann–Whitney U test. The non-parametric effect size *r* was calculated as *Z*/√N. Von Frey data represent the percentage decrease in mechanical paw withdrawal threshold from the pre-fracture baseline. Temperature difference is the asymmetry (°C) between the ipsilateral (fractured) and contralateral hind paws. Edema is expressed as the percentage increase in thickness of the ipsilateral paw relative to the contralateral paw.

**Table 2:** Inflammatory Cytokine Levels in Ipsilateral Hind Paw Tissue Homogenates.

Parameter	Control	ASU	*p*-Value	Effect Size
IL-1β (pg/mg protein)	59.84 [52.32–65.20]; 59.82 ± 9.16	34.90 [31.13–37.09]; 34.54 ± 3.62	<0.001	0.84
IL-6 (pg/mg protein)	82.39 [74.29–91.51]; 81.02 ± 13.55	40.25 [36.04–44.15]; 39.98 ± 5.40	<0.001	0.84
TNF-α (pg/mg protein)	52.02 [48.84–57.85]; 52.86 ± 8.39	22.12 [19.82–24.35]; 22.31 ± 2.96	<0.001	0.84

Data are presented as median [interquartile range, IQR] and mean ± standard deviation (SD) of cytokine concentrations in ipsilateral hind paw tissue homogenates, expressed as picograms per milligram of total protein (pg/mg protein). Group comparisons were performed using the Mann–Whitney U test. The non-parametric effect size *r* was calculated as *Z*/√N. All comparisons showed statistical significance at *p* < 0.001.

**Table 3:** Oxidative Stress Parameters in Ipsilateral Hind Paw Tissue Homogenates.

Parameter	Control (Median [IQR]; Mean ± SD)	ASU (Median [IQR]; Mean ± SD)	*p* Value	Effect Size (r)*
TAS (mmol Trolox equation/mg protein)	1.36 [1.16–1.40]; 1.33 ± 0.17	2.06 [1.92–2.29]; 2.12 ± 0.23	<0.001	0.84
TOS (µmol H_2_O_2_ equation/mg protein)	17.32 [14.15–17.77]; 16.44 ± 2.12	11.20 [8.46–12.03]; 10.65 ± 1.95	<0.001	0.80
OSI (AU)	1272.18 [973.90–1531.90]; 1262.93 ± 271.69	488.49 [425.91–605.11]; 507.82 ± 104.16	<0.001	0.84

Data are presented as median [interquartile range, IQR] and mean ± standard deviation (SD). TAS: total antioxidant status, expressed as mmol Trolox equivalent per mg of total protein. TOS: total oxidant status, expressed as μmol H_2_O_2_ equivalent per mg of total protein. OSI: oxidative stress index, calculated as (TOS/TAS) × 100, expressed in arbitrary units (AU). Group comparisons were performed using the Mann–Whitney U test. The non-parametric effect size *r* was calculated as *Z*/√N. * Non-parametric effect size (r), calculated as Z/√N.

## Data Availability

Additional data are available from the corresponding author upon reasonable request.

## Abbreviations

The following abbreviations are used in this manuscript:

CRPS-I	Complex Regional Pain Syndrome Type I
ASU	Avocado/Soybean Unsaponifiables
TAS	Total Antioxidant Status
TOS	Total Oxidant Status
OSI	Oxidative Stress Index
IL	Interleukin
TNF-α	Tumor Necrosis Factor Alpha
ELISA	Enzyme-Linked Immunosorbent Assay
ARRIVE	Animal Research: Reporting of In Vivo Experiments
